# Berlin Letter

**Published:** 1891-05

**Authors:** Julius Pohlman

**Affiliations:** Berlin


### Correspondence.

### BERLIN LETTER.

*Letter from Prof. Pohlman— The Twentieth Congress of German Surgeons— Opinions of Von Bergman, Schede, Liebreich, and others— Guajacol, Cantharidis, and Iodoform in Tuberculosis.*

THE Twentieth Congress of Surgeons opened its meeting here on April 1st. After a careful and deliberate review, the Executive Committee determined upon a discussion of that all-absorbing topic, Koch's lymph, for the first day of its meeting, and the following is a short *résumé* of it :

The President, Prof. Von Bergman, Berlin, stated that the characteristic local re-action following the injection of tuberculine occurs only in tubercular cases, but the general re-action has been observed in cases of aktinomykosis and in large soft sarcomæ. In the latter, the possibility that the patients were also suffering from some tubercular trouble was by no means excluded, and careful post-mortems should always be made to determine the exact conditions. It is well known that patients suffering with local tubercular lesions, when subjected to severe surgical operations occasionally develop all the symptoms of general tuberculosis. We may compare the injection of Koch's lymph to such a surgical operation, and thus explain the cases on record where miliar-tuberculosis appeared after an injection, and it will be necessary to demonstrate first by the most careful post-mortem the presence of only one localized tubercular nodule before we blame Koch's method for the production of general tuberculosis. It should also be demonstrated that the spreading of the bacilli was recent and had been induced by the tuberculine, and that demonstration will probably forever remain an impossibility. He had seen many cases where, after what appeared to be a complete recovery, new lesions appeared in places not affected before, and the explanation given had been that they represented latent nodules ; but as they did not put in their appearance until six to ten weeks after the treatment, this explanation had its objections. More objectionable yet is the assumption that these so-called latent nodules were not affected by the tuberculine, because it is always claimed that *recent* cases are influenced easiest. Certain it is that the tuberculine confers no immunity to the human body against the attacks of the bacillus, nor does it protect against a relapse.

The time of observation had been too short to determine whether the treatment by tuberculine should be modified or rejected, and physicians would have to learn constantly while using it, and record their observations for the benefit of medical science.

Prof. Koenig, of Göttingen, agreed with Von Bergman as to the certainty of the local re-action, and that the remedy should be used with the greatest care. His results had been more favorable since he commenced with small doses,—one-half milligram. His opinion of the treatment was decidedly hopeful. He had also tried iodoform injections, like Von Bergman, with the same negative results.

Schede, of Hamburg, looked upon tuberculine as the most valuable weapon known today to fight tuberculosis. He had never seen any serious symptoms following its use, but also advocated the use of very small doses to prevent fever as much as possible, and puts special stress upon the necessity of proper food, fresh air, hygienic surroundings, etc.

Lauenstein, of Hamburg; Von Eiselsberg, of Billroth's Clinic, in Vienna; and Kuester, of Marburg, all agree that tuberculine is a very useful therapeutic agent if care has been taken in selecting the cases to be teated.

Dr. Senger, of Crefeld, reports that in surgical tuberculosis, a valuable drug had been found in iodoform, but only in the treatment of tubercular joints. He claims that it is not the iodoform itself which acts, but the formic acid produced by the oxidation of the iodoform. Hence, if oxidation can take place, the iodoform acts, but not otherwise.

Dr. Rubinstein reports for Prof. Schüler, of Berlin, the record of 100 cases of localized gland, bone or joint tuberculosis treated with guajacol, and with a combination of surgical treatment and iodoform injections. The anti-tubercular action of guajacol, the active principle of creosote, had been demonstrated upon animals long ago, and out of fifteen patients treated by it, nine cases were cured, five are yet under treatment, and only one had to be discontinued.

The recently reported discovery of Prof. Liebreich, for the cure of tuberculosis, cantharidis, was discussed by Von Bergman, who stated that in his clinics the new remedy had been used in several cases of lupus, but without any results. Prof. Liebreich claimed on the other hand that a slow improvement *had* taken place in the condition of these patients, to which Von Bergman replied that these apparent improvements were nothing else than the peculiar changeable conditions always found more or less in patients suffering from lupus. Liebreich expressed the hope that time would determine the value of his remedy better than a discussion.

The paper I send you herewith[1] I have translated for the JOURNAL, as it, perhaps, represents the most condensed review of the tuberculine question. Dr. Thorner is one of the first six physicians to whom Dr. Koch gave a bottle of the lymph for trial, and he has been using it in a large private practice ever since.

JULIUS POHLMAN.

BERLIN, April 3, 1891.

1. Published on page 582 *et seq.*

